# Effect of Orthodontic Movement on the Periapical Healing of Teeth Undergone Endodontic Root Canal Treatment: A Systematic Review

**DOI:** 10.3390/jcm14207292

**Published:** 2025-10-16

**Authors:** Hanan Alharbi, Mishal S. Almutairi, Suliman Alrajhi, Nabeel Almotairy

**Affiliations:** 1 Department of Conservative Dental Sciences, College of Dentistry, Qassim University, Qassim, Saudi Arabia; 2 Private Sector, Qassim 52391, Saudi Arabia; 3 Saudi Board of Orthodontics, Qassim Regional Dental Center, Buraydah 52391, Qassim, Saudi Arabia; 4 Department of Orthodontics and Pediatric Dentistry, College of Dentistry, Qassim University, Buraydah 52571, Qassim, Saudi Arabia; n.s.almotairy@qu.edu.sa

**Keywords:** root canal treatment, orthodontic movement, periapical healing, tooth, treatment quality, endodontics

## Abstract

**Background:** The relationship between orthodontics and endodontics during the treatment planning phase is scarcely investigated, especially when orthodontic treatment is considered for endodontically treated teeth with apical periodontitis. This systematic review aimed to investigate the effect of orthodontic movement on the periapical healing of teeth that have undergone endodontic root canal treatment/retreatment. **Materials and methods:** On 15 March 2025, a systematic search was conducted in PubMed, Web of Science Core Collection, EBSCO host and complemented with a manual search of Google Scholar and the gray literature. The quality and the risk of bias of the included studies were assessed using the Joanna Briggs Critical Appraisal tools for human studies and the Systematic Review Centre for Laboratory Animal Experimentation for animal studies. The data about the influence of orthodontic movement on the periapical healing of endodontically treated teeth were extracted and pooled. **Results:** Out of 4614 identified titles, 6 studies were finally included (two animal and four clinical studies). The risk of bias was high in one study, moderate in three, and low in two. The included animal studies demonstrated a significant delay in the healing process of periapical lesions when orthodontic forces were applied shortly after root canal treatment. However, clinical studies showed no significant impact of orthodontic movement on periapical radiolucency except when the quality of obturation was compromised. **Conclusions:** Current clinical studies indicate that orthodontic tooth movement does not impair the periapical healing of endodontically treated teeth when the root canal obturation is of adequate quality.

## 1. Introduction

The interplay between endodontics and orthodontics is a crucial part of the treatment plan, highlighting the intricate relationship between the outcomes of both disciplines. Understanding this relationship is critical for achieving optimal clinical prognosis and ensuring comprehensive patient care. Previous research has predominantly centered on the implications of orthodontic forces on root resorption [1], the effects of orthodontic movement on traumatized teeth [2], and the response of vital pulp to such treatments [3,4]. Root resorption has been a focal point, with studies revealing that both vital and endodontically treated teeth (ETT) are susceptible to resorptive processes when subjected to orthodontic forces [5]. Overall, while orthodontic forces induce a range of inflammatory and metabolic changes in the periodontal tissues and pulp, these effects are generally transient and reversible, provided the forces are within a physiologically acceptable range [6].

The relationship between orthodontic treatment and the healing process of endodontically treated teeth has received relatively limited attention. Despite its potential significance in clinical outcomes, this area still needs to be explored. The primary concern is whether the application of orthodontic forces might delay the periapical healing or not. Early studies on dogs demonstrated that orthodontic movement applied shortly after endodontic treatment significantly delayed healing, indicating a possible destabilization of the healing environment within the alveolar bone [7,8]. Recently, the literature has delved into the radiographic outcomes associated with such treatments. These studies highlight that the quality of the endodontic treatment might play a pivotal role in determining the periapical response to subsequent orthodontic forces. A retrospective analysis found that teeth with poor initial endodontic treatment quality were more susceptible to periapical bone destruction after orthodontic intervention, whereas adequately treated teeth displayed minimal adverse effects, emphasizing the importance of meticulous endodontic procedures prior to orthodontic therapy [9]. Others found no clear correlation between changes in the size of periapical radiolucency of ETT and orthodontic treatment [10,11].

Periapical radiolucency is a critical radiographic finding used to detect and follow up on apical periodontitis (AP). The detection sensitivity of AP is known to be significantly higher in computed cone-beam computed tomography (CBCT) compared to periapical radiographs [12]. AP is prevalent mainly in posterior molars [13], where those teeth are frequently used as anchorage units in orthodontic therapy. Despite the growing prevalence of orthodontic treatment in adult populations and the frequent involvement of endodontically treated teeth, there is no clear consensus on whether orthodontic forces adversely affect the periapical healing process. This systematic review aims to investigate the effect of orthodontic movement on the periapical healing of teeth that have undergone endodontic root canal treatment/retreatment.

## 2. Materials and Methods

### 2.1. Protocol and Registration

This systematic review’s protocol was registered in the Open Science Framework Repository “https://osf.io/jnzm6 (accessed on 20 July 2024)”. The recent update of the PRISMA guidelines was followed when conducting this systematic review [14].

### 2.2. Information Sources and Search Strategy

On 15 March 2025, a comprehensive search was initiated by an experienced author on four databases: PubMed, Web of Science Core Collection, EBSCO host (MEDLINE, Academic Search Ultimate, ERIC, Library, and Information Science & Technology Abstracts), and ProQuest. The database search strategy was first conducted on PubMed and then adapted to other databases (Table 1). A manual search of Google Scholar was also conducted to complement the database search, where the first 100 results were sorted based on relevance and screened for eligible studies. In addition to a search in the grey literature “www.opengrey.eu/ (accessed on 15 March 2025)”. No restrictions were placed on the study publication date, type, or language during the database search.

### 2.3. Study Eligibility Criteria

The current systematic review applied the following population, intervention, comparison, and outcome (PICO) question:

- Population: Living humans or animals with endodontically treated teeth.
- Intervention: Orthodontic tooth movement.
- Comparison: Controls (no orthodontic tooth movement or baseline status).
- Outcome: Periapical healing status (radiographic/histologic).

The study inclusion criteria were as follows:

1. Studies of living humans or animals examining the influence of orthodontic tooth movement on the periapical healing of teeth undergone root canal treatment/retreatment.
2. Success of root canal treatment is based on clinical and/or radiographic criteria (strict = absence of apical radiolucency; loose = reduction in size of radiolucency).
3. Overall success rate was assessed or could be calculated from the raw data.

While the study exclusion criteria were:

1. Studies investigating the influence of orthodontic tooth movement on root resorption or endodontically teeth with a trauma history.
2. Other types of publications, include case reports/series, abstracts, reviews, editorials, letters to editors, and author opinion papers.
3. Studies written in a language other than English.

### 2.4. Study Selection

The database search result was exported to Mendeley software (version 2.116.0, Elsevier, London, UK), where the duplicates were removed by the built-in duplicate removal feature and manually confirmed for accuracy. Then, the de-duplicated result was exported to Microsoft Excel, where two reviewers (M.A. and S.A.) independently screened the study’s title/abstract and classified them based on our inclusion/exclusion criteria to included, excluded, or undecided studies. Undecided studies were resolved by mutual discussion or by consulting senior authors (H.A. and N.A.). Then, the full text of the initially included studies was independently examined by the two authors to further classify them into included, excluded, or undecided. Again, the decision to include the studies was resolved by a mutual discussion and/or consulting the senior authors. The study selection timeframe was from 16 March 2025 till 15 April 2025.

### 2.5. Quality Assessment and Data Extraction

The methodological quality assessment of the included studies was conducted by the two independent authors using the Joanna Briggs Critical Appraisal tools (JBI) for human studies [15] and the Systematic Review Centre for Laboratory Animal Experimentation (SYRCLE) for animal studies [16]. The two reviewers (M.A. and S.A.) underwent internal calibration sessions using sample articles to ensure consistent application of the JBI critical appraisal tools. The cumulative score of each study’s quality assessment was calculated by assigning one mark for a positive response (Yes) to the instrument questions and zero for other responses (No or Unclear). Then, the percentage of positive responses to the total number of questions was calculated, and the study was subsequently rated either high (80–100%), moderate (51–79%), or low (equal to or less than 50%) quality based on the resultant percentage. Disagreements in the quality assessment were resolved by a mutual discussion and/or consulting the senior authors.

The two authors extracted data from the included studies. The data were as follows: (1) Author(s), study methodology, country, and year of publication; (2) Sample characteristics (age and gender); (3) Teeth sample (number and type); (4) Malocclusion type for human studies; (5) Endodontic treatment procedure; (6) Orthodontic treatment type and duration; (7) Periapical assessment method; (8) Outcome of interest; and (9) Main study results. Due to the heterogeneity across the included studies, a quantitative meta-analysis was not feasible. Therefore, the findings were synthesized narratively.

## 3. Results

**Figure 1:**
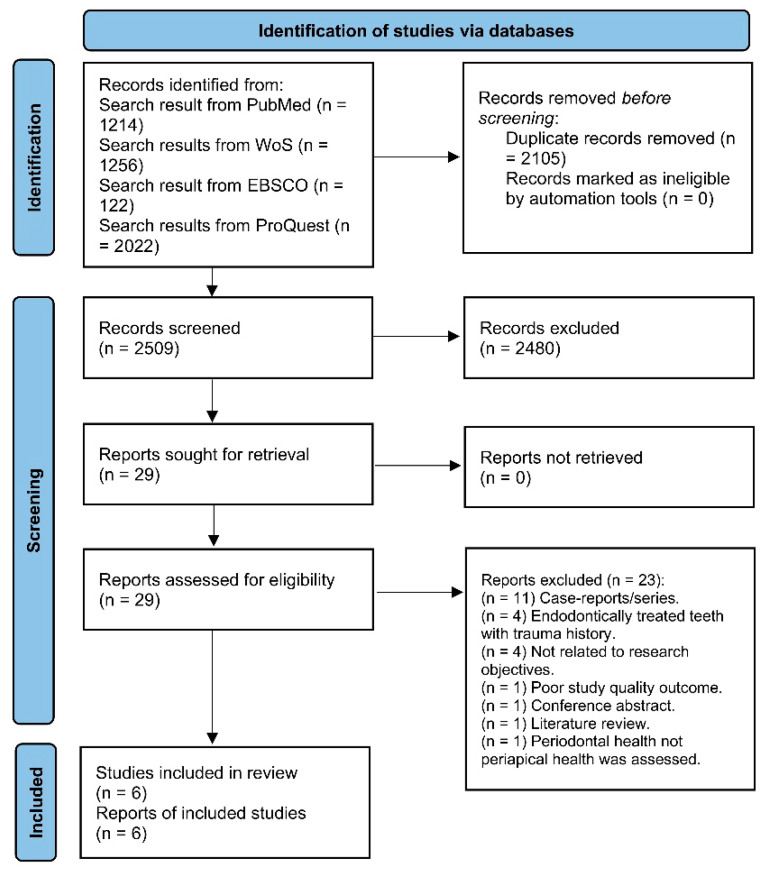
PRISMA flow diagram of study screening and selection process.

A total of 4614 studies were retrieved from the database search (Figure 1). After removing the duplicates, 2509 studies remained for the article/abstract screening process, where 2480 studies were excluded. The full text screening of the remaining 29 studies yielded twenty-three excluded studies for various reasons (Appendix A). After the screening process, a list of 6 studies satisfying our PICO question was included in the current systematic review [7,8,9,11,17,18].

### 3.1. Study Characteristics

Table 2 presents detailed characteristics of the included studies. Four studies were retrospective, three cross-sectional and one cohort in design involving living human participants [9,11,17,18], while the other two studies were cross-sectional animal studies, each were employing two dog samples [7,8]. The sample size of the four clinical studies ranges from 61 to 100 participants, with diverse age groups from adolescence to late adulthood [9,11].

Concerning the endodontic treatment procedure, three clinical studies reported that root canal treatment was conducted without further details [9,11,18]. Th fourth one reported using laser-activated irrigation (Er:YAG) during root canal treatment [17]. For the animal studies, de Souza et al. induced a periapical lesion by exposing the root canals for six months, followed by root canal treatment of investigated teeth [8], while Baranowskyj performed root canal treatment with apicectomy with no further details regarding the induction of periapical lesions [7].

Regarding the quality of root canal obturation, all four clinical studies assessed obturation using radiographic parameters, though with different classification systems. Kim et al. evaluated the quality using two standards: (1) distance from the apical end of the root to the canal obturation and materials and (2) presence of voids (65.3% good, 15.7% moderate, 15.7% poor, and 3.5% no canal obturation) [11]. Similarly, Chen et al. considered fillings adequate if they ended within 0–2 mm of the apex with no voids (70.3%); inadequate fillings (41.5%) included over-extended, underfilled, or poorly condensed canals [18]. Alqerban et al. assessed RCT quality using length, density, and homogeneity [9]. The RCT quality was scored as adequate for 105 (82%), unsure for 7 (5%), and inadequate for 16 (13%) teeth. Ianos et al. used Alqerban’s classification relying on both length (66% correspondent, 3% uncertain, 31% inadequate) and density/homogeneity (78% correspondent, 6% uncertain, 16% inadequate) [17]. Two studies evaluated the coronal restoration of ETT teeth and found it adequate in 66.9% to 95% of their samples [9,18]. Across all studies, inadequate root fillings were consistently or significantly associated with higher rates of periapical lesion progression following orthodontic treatment.

Orthodontic tooth movement influence on the periapical healing was radiographically assessed using orthopantomograms [9] or cone-beam computed tomography [11,17,18] in the four clinical studies. While the animal studies sacrificed the sample dogs and histologically assessed the periapical healing [7,8]. Alqerban et al. used two indices to assess the degree of periapical bone destruction, the Periapical 1 (PAI) and the Probability Index (PRI) [9]. Ianos et al. [17] used PRI index only and Chen et al. used CBCT periapical index [18,19]. There was no significant increase in PAI and PRI scores after orthodontic treatment for adequately treated teeth [9,17]. The other two CBCT studies were different, one demonstrated a significant association between the CBCT periapical index and RCT quality [18], while the other found no significant effect [17]. Significant reductions in lesion size were more frequently observed in female patients and in maxillary teeth [11]. Animal studies revealed a significant reduction in lesions [8] and significantly better healing [7] in non-orthodontic groups without further details related to the size.

The malocclusion complexity was not clearly reported in the four clinical studies. However, all used fixed orthodontic appliances with a diverse treatment duration ranging from one year to more than four years [9,11] or unspecified durations [17,18]. Short-term fixed orthodontic appliances were used in the animal studies to apply orthodontic forces on the investigated teeth; five months with fifteen days [8] and two weeks [7]. Periodontal condition was assessed radiographically based on bone loss severity, but no significant effect on periapical lesion changes was observed during orthodontic treatment [18]. RCT completion in relation to the start of orthodontic tooth movement was not mentioned in two studies [11,18]; however, three studies reported that it was done before orthodontic tooth movement, either two weeks [7], immediately [8], or with no specific time-frame [9]. While the remaining study reported that RCT was completed during orthodontic tooth movement [17]. For the force magnitude of orthodontic movement, only one study reported such information, stating a 200 g force application [8].

### 3.2. Study Quality Assessment

**Figure 2:**
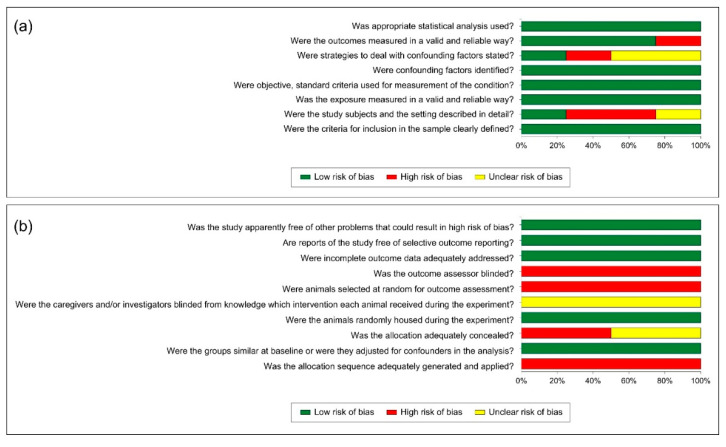
Risk of bias result of the included studies using the JBI (**a**) and SYRCLE (**b**) assessment tools.

Figure 2 shows the risk of bias assessment using the JBI (Figure 2a) and SYRCLE tools (Figure 2b). The quality assessment of the included studies revealed that two study had a low risk of bias [11,18], three were moderate [8,9,17], and one had a high risk of bias [7]. Overall, animal studies showed high or unclear risks concerning the sample allocation sequence, concealment, and random selection. They also showed high or unclear risk of bias concerning the blindness of investigators and outcome data assessment. However, they had adequately addressed sample baseline similarity and were free from other problems that could increase the risk of bias. For human studies, they appropriately addressed all JBI tool criteria, except the detailed description of study participants and handling of confounding factors. Specifically, one study demonstrated high or unclear risk due to reliance on orthopantomograms for periapical assessment [9], whereas CBCT-based studies [11,17,18] provided more reliable outcome measurements. Due to the high heterogeneity among the included studies, a meta-analysis was not conducted.

### 3.3. The Outcome of the Included Studies

The included studies showed that orthodontic tooth movement of teeth undergone root canal treatment can delay the healing process of periapical hard and soft tissues [7,8,11] without hindering the healing process. The radiographic assessment revealed that the size of the periapical lesion remained unchanged after orthodontic fixed appliance treatment [9,11]. Traction distance and rotation angle measured by CBCT superimposition showed no significant effect on periapical lesion progression during orthodontic treatment [18]. However, it was greatly influenced by the quality of root canal treatment, where teeth with inadequate root canal treatment exhibited a greater risk of further periapical bone destruction and lesions [9,17,18]. Additionally, patient’s gender and tooth type were also associated with the degree of orthodontic tooth movement influence on root canal-treated teeth. Specifically, Kim et al. showed that female patients and maxillary teeth exhibited a greater reduction in periapical lesion of root canal-treated teeth after orthodontic fixed appliance treatment compared to males and mandibular teeth, respectively, [11].

## 4. Discussion

The relationship between orthodontics and endodontics during treatment planning phase is scarce, especially when orthodontic treatment is considered for ETT with AP. Thus, this systematic review evaluated the effect of orthodontic treatment on the periapical healing of teeth that had undergone endodontic root canal treatment (RCT) or retreatment. Six studies met the inclusion criteria, consisting of four human and two animal studies, revealing heterogenic findings related to healing patterns, the influence of root canal treatment quality, orthodontic treatment period, tooth movement parameters, and the impact of patient demographics. The primary result drawn from the included studies, particularly the human studies, is that periapical healing outcomes are predominantly determined by the quality of the root canal filling rather than by the extent of orthodontic tooth movement, rotation, or periodontal condition. Orthodontic tooth movement may transiently delay healing in experimental animal models [7,8]; however, clinical studies with larger and more representative sample sizes consistently indicate that, when root canal treatment and coronal restoration are of high quality, orthodontic treatment mostly does not adversely affect periapical healing [6,9,11,17,18]. These findings provide important insights into the complex relationship between orthodontic forces and endodontically treated teeth, specifically regarding the size of periapical radiolucency.

The included animal studies [7,8] demonstrated a significant delay in the healing process when orthodontic forces were applied shortly after RCT. Baranowskyj found that orthodontic tooth movement applied within two weeks of RCT, combined with apicectomy, delayed the healing of soft and hard tissues. Similarly, De Souza et al. [8] observed delayed periapical healing in dogs after five months and 15 days of orthodontic treatment, although healing was not completely obstructed. On the contrary, human studies [9,11,17,18] found no significant effect of orthodontic tooth movement on the size of periapical radiolucency in endodontically treated teeth, except when the quality of the initial RCT was compromised [9,17,18]. The discrepancies between animal and human studies occur primarily from methodological, temporal, and biological differences. While animal models allow detailed observation of early cellular and inflammatory changes via histological analysis, human studies are often limited to long-term radiographic findings.

The observed delay in healing can be explained by the complex biological processes at the periapical region during orthodontic movement. Orthodontic forces induce inflammatory and metabolic changes in the periodontal ligament (PDL), which could interfere with the normal healing processes of periapical tissues [20]. These inflammatory responses, although transient, can alter the blood flow and cellular environment around the tooth, potentially destabilizing an already healing periapical lesion [6]. This concept is particularly relevant when orthodontic forces are applied too soon after endodontic treatment, as seen in the animal studies included in this review. This reflects that while biological plausibility for delayed healing exists and demonstrated in animal histological models, clinical studies relying on radiographic outcomes may not fully capture such subtle early changes. Orthodontic forces are known to induce osteoclast-mediated bone remodeling and transient inflammatory responses, which may interfere with early stages of periapical tissue repair, but such microscopic alterations are rarely detectable in radiographic human studies [21].Thus, it is recommended that orthodontic treatment be initiated after a period of 15 to 30 days on endodontically treated teeth, regardless of the presence or absence of AP [22].

Animal dental studies differ from clinical studies primarily in the accuracy and depth of histological data, which allows for detailed observation of cellular-level changes, inflammatory responses, and the precise extent of tissue healing or damage. Clinical studies in this review, which rely on radiographic methods, are non-invasive and valuable for longitudinal follow-up, allowing the visualization of broader anatomical changes such as bone density or lesion size. However, they are limited by their resolution and susceptibility to artifacts, potentially overlooking microstructural details [23]. CBCT enhances radiographic assessment by providing three-dimensional images and high-spatial-resolution of complex dental structures and periapical lesions, which are often challenging to visualize with two-dimensional radiographs [19,24]. Thus, while animal studies reported significant effects of orthodontic forces on periapical healing, findings from human CBCT-based studies suggest that such effects may not be clinically relevant when root canal treatment quality is optimal.

Radiographs are pivotal for the diagnosis and follow-up of apical periodontitis. Periapical radiographs (PAs) are routinely used due to their accuracy and superior resolution compared to panoramic images [25]. However, the reproducibility of these two-dimensional radiographs relies heavily on consistent angulation and patient positioning. It is well known that variations in the horizontal and vertical angulation of the X-ray beam can lead to distortions and inaccurate assessments of periapical lesions [26], making follow-up assessments less reliable. The paralleling technique in PAs is generally recommended as it minimizes image distortion, ensuring more consistent and accurate measurements across different follow-ups [27]. Additionally, with an average of 1.8 teeth per patient requiring follow-up radiographs [9,11], PAs are the preferred choice for monitoring apical periodontitis compared to large-scale panoramic images. CBCT, on the other hand, has greater sensitivity to detect AP than panoramic and PAs [12]. According to the ALARA principle, “as low as reasonably achievable,” limited-view CBCT should be chosen to follow up periapical lesions instead of large-scan CBCT [28]. In Kim et al. study [11], the largest linear distance (mm) was used as the size of the periapical radiolucency index, where most studies usually assess changes in the total volume of periapical lesions.

The quality of the endodontic treatment emerges as the critical factor influencing periapical healing during orthodontic movement. Teeth with substandard root canal fillings or persistent periapical lesions before orthodontic treatment exhibit worse outcomes [9,17,18]. This is consistent with studies on endodontic outcomes that have always emphasized the importance of RCT quality in determining periapical health prognosis [29]. The outcome of periapical status depends on many factors, including steps in chemo-mechanical debridement [29] and achieving a coronal and apical seal [30]. RCTs are evaluated radiographically using the level and density of the root filling, with an optimal obturation level being 0–2 mm from the radiographic apex [31,32]. Therefore, finalizing coronal restoration before initiating orthodontic therapy is a must. Complete healing of periapical lesions can occur within 1 year, however, it’s possible for healing to take several years [29] regardless of other interventions like orthodontic treatment.

Gender and tooth type are known to have no significant effect on RCT success and periapical healing [29,32]. The more significant reduction of periapical lesions in female patients compared to male patients in Kim’s study can be attributed to differences in included samples (76F:39M) [11]. The same study reported that mandibular teeth had less periapical lesion reduction compared to maxillary teeth. This is in accordance with some other studies where mandibular teeth had the lower success rate compared with other tooth types [33,34]. The initial size of periapical lesion significantly affects the healing process, where the smaller the size, the better the treatment prognosis. This should be carefully considered when commencing orthodontic treatment in cases with large periapical radionuclides (≥5 mm) [29,35].

### Limitations

There are several limitations that should be carefully considered when interpreting the current study’s results. A prime limitation of this review is that it included a small number of studies with varying methodologies, sample sizes, and assessment techniques that can limit the generalizability of the results. Such assessment techniques of periapical radiolucency size included histological analysis, panoramic radiographs, and/or CBCT imaging. Moreover, two of the included studies were animal-based, and while animal models provide valuable insights, they may not fully replicate the human biological environment. Additionally, the included clinical studies did not control known factors that can influence periapical outcomes during orthodontic treatment. For example, previous literature has shown that pre-orthodontic malocclusion type, tooth movement distance and duration, orthodontic force type and magnitude, type of teeth to be moved, and previous orthodontic history can significantly influence tooth apical tissue response [36,37,38]. All in all, those variables can affect the consistency and accuracy of the reported outcomes and necessitate planning controlled clinical trials with larger sample sizes that take into consideration the above limitations to better investigate the topic. Based on the available evidence, it is recommended to ensure high-quality root canal treatment with adequate coronal sealing before initiating orthodontic forces. Although orthodontic treatment may transiently delay periapical healing, it does not appear to adversely affect ultimate healing outcomes when endodontic treatment is performed to a high standard.

## 5. Conclusions

Within the limitations of the available evidence, current clinical studies indicate that orthodontic tooth movement does not appear to impair the periapical healing of endodontically treated teeth when the root canal treatment is of adequate quality. High-quality root canal treatments appear to mitigate the adverse effects, emphasizing the need for meticulous endodontic procedures before orthodontic intervention. This systematic review highlights the importance of understanding the complex relationship between orthodontic forces and the healing of periapical tissues in endodontically treated teeth.

## Figures and Tables

**Table 1:** The database search strategy.

**PubMed**
((Orthodontics, Corrective * [MeSH Terms]) OR (Orthodontic * [Title/Abstract] OR Tooth movement [Title/Abstract])) AND ((Apical periodontitis [Title/Abstract] OR Periapical periodontitis [Title/Abstract] OR Apical lesion * [Title/Abstract] OR Periapical lesion * [Title/Abstract] OR Root-filled tooth [Title/Abstract] OR Root-filled teeth [Title/Abstract] OR Root canal treatment [Title/Abstract] OR Endodontic * [Title/Abstract] OR Periapical abscess [Title/Abstract] OR Apical abscess [Title/Abstract] OR Periapical cyst * [Title/Abstract] OR Apical cyst * [Title/Abstract]) OR (Periapical Periodontitis/physiopathology * OR Periapical Periodontitis * OR Root Canal Therapy OR Root Canal Obturation))
**Web of Science Core Collection**
(TS = (Orthodontic * OR Tooth movement)) AND TS = (Apical periodontitis OR Periapical periodontitis OR Apical lesion * OR Periapical lesion * OR Root-filled tooth OR Root-filled teeth OR Root canal treatment OR Endodontic * OR Periapical abscess OR Apical abscess OR Periapical cyst * OR Apical cyst *) Indexes = SCI-EXPANDED, SSCI, A&HCI, CPCI-S, CPCI-SSH, ESCI Timespan = All years
**EBSCO host (MEDLINE, Academic Search Ultimate, ERIC, Library, and Information Science & Technology Abstracts)**
(TI (Apical periodontitis OR Periapical periodontitis OR Apical lesion * OR Periapical lesion * OR Root-filled tooth OR Root-filled teeth OR Root canal treatment OR Endodontic * OR Periapical abscess OR Apical abscess OR Periapical cyst * OR Apical cyst *) OR MH (Periapical Periodontitis/physiopathology * OR Periapical Periodontitis * OR Root Canal Therapy OR Root Canal Obturation)) AND (TI (Orthodontic * OR Tooth movement) OR MH (Orthodontics, Corrective *))
**ProQuest**
(mainsubject(Periapical Periodontitis/physiopathology * OR Periapical Periodontitis * OR Root Canal Therapy OR Root Canal Obturation) OR abstract(Apical periodontitis OR Periapical periodontitis OR Apical lesion * OR Periapical lesion * OR Root-filled tooth OR Root-filled teeth OR Root canal treatment OR Endodontic * OR Periapical abscess OR Apical abscess OR Periapical cyst * OR Apical cyst *) OR title(Apical periodontitis OR Periapical periodontitis OR Apical lesion * OR Periapical lesion * OR Root-filled tooth OR Root-filled teeth OR Root canal treatment OR Endodontic * OR Periapical abscess OR Apical abscess OR Periapical cyst * OR Apical cyst *)) AND (mainsubject(Orthodontics, Corrective *) OR abstract(Orthodontic * OR Tooth movement) OR title(Orthodontic * OR Tooth movement))

**Table 2:** Detailed characteristics of the included studies.

Citation	Study Type; Country; Date Publication	Sample Size (Age); Gender Distribution	Investigated Teeth	Malocclusion Type	Endodontic Treatment Type	Timing of Endodontic Treatment	Orthodontic Treatment Type; Duration	Orthodontic Forces	Periapical Assessment Method	Outcome of Interest	Main Results	Study Quality
(Alqerban et al., 2019) [9]	Cross-sectional retrospective clinical study; Saudi Arabia; 2019	72 patients (23.4 ± 6.9 yr); 49 F: 23 M	128 teeth (incisors, premolars, and molars)	Not mentioned.	Primary RCT but no further details	Before orthodontic tx.	FA but no further details; tx duration 1.8 ± 1.1 yr	Not mentioned.	OPG assessment of RCT quality, length, density, and homogeneity, and coronal restoration. The probability index and the periapical index were used to evaluate periapical bone destruction and healing, respectively before and after orthodontic treatment.	RCT treatment integrity and quality, and periapical bone destruction and lesions.	Teeth with adequate RCT showed no changes in the periapical bone destruction or healing after orthodontic tx, while teeth with inadequate RCT had greater risk of periapical bone destruction and lesions after orthodontic tx.	Moderate
(Kim et al., 2023) [11]	Cross-sectional retrospective clinical study; South Korea; 2023	61 patients (14–54 yr); Not clear	115 teeth (37 anterior teeth, 22 premolars and 56 molars)	Not mentioned.	RCT but no further details	Not mentioned.	Not reported; tx duration was variable with 24 mo and more than 48 mo	Not mentioned.	CBCT was used to evaluate the size of the periapical radiolucency of endodontically treated teeth before and after orthodontic tx.	The size of periapical radiolucency	Orthodontic treatment did not affect the size of periapical radiolucency. However, females and maxillary teeth showed statistical reduction compared to males and mandibular teeth, respectively.	High
(De Souza et al., 2006) [8]	Cross-sectional animal study; United States; 2006	2 dogs (1 yr); not mentioned	Incisors and premolars	Not applicable	Root canals were kept opened for 6 mo to induce periapical lesion followed by RCT	Immediately before orthodontic movement.	FA with power chain; tx duration 5 mo and 15 d	200 g	Animals were scarified and 10 specimens for 16 histological criteria were taken from RCT-free teeth that moved orthodontically, RCT-teeth with no orthodontic movement, and RCT-teeth that moved orthodontically.	Healing of periapical lesions.	The results showed that orthodontic forces delayed but did not hinder the periapical healing process.	Moderate
(Baranowskyj, 1969) [7]	Cross-sectional animal study; United States; 1969	2 dogs (not mentioned); females	Maxillary second incisors	Not applicable	RCT and apicectomy 6 w apart	2 w before orthodontic treatment.	Orthodontic intrusive forces for 4 w.	Not mentioned.	Animals were scarified and histological investigation was conducted of periapical healing of both control and experimental dogs.	Healing of periapical tissues.	Early application of orthodontic forces on teeth undergone RCT can delay the healing process of periapical soft and hard tissues.	Low
(Chen et al., 2024) [18]	Retrospective cohort clinical study; China; 2024	169 teeth of 100 (52 F + 48 M) age (33–23) median age 27.	Mandibular teeth (63) Maxillary teeth (44) Anterior teeth (27) Premolars (37) Molars (43).	Not mentioned.	Primary RCT.	Not mentioned.	Fixed orthodontic movement ortho treatment was 2.6 years.	Not mentioned.	Pre-orthodontic and post-orthodontic CBCTs Absence of AP was assigned 0, while the presence of AP > 0.5 was assigned a score from 1 to 5 based on severity.	Healing of periapical tissue.	Orthodontic movement had no effect on the outcomes of RCT teeth.	High
(Ianos et al., 2024) [17]	Cross-sectional retrospective clinical study; Romania; 2024	32 teeth from 25 patients.	Not mentioned.	Not mentioned.	Primary RCT.	During orthodontic tx.	Fixed orthodontic movement.	Not mentioned.	CBCT Periapical bone destruction probability index (PRI) was evaluated as follows (absent, uncertain or present).	The size of apical radiolucency.	Endodontically teeth with inadequate RCT show a significantly higher risk of periapical bone destruction.	Moderate

**Abbreviations:** CBCT, Cone Beam Computed Tomography; d, days; F, females; FA, fixed orthodontic appliance; g, gram; M, males; mo, months; OPG, orthopantomogram; RCT, root canal treatment; tx, treatment; w, weeks; yr, years.

## Data Availability

Data are available upon requested.

## Appendix A. The List of the Excluded Studies Based on the Full Text Assessment with the Reasons of Exclusion

**No.**	**Citation**	**Reason of Exclusion**
1	Ozalp, S. O., Tuncer, B. B., Tulunoglu, O., & Akkaya, S. (2008). Endodontic and orthodontic treatment of fused maxillary central incisors: a case report. *Dental Traumatology*, *24*(5). https://doi.org/10.1111/j.1600-9657.2008.00635.x	Endodontically treated teeth with a trauma history
2	Wickwire, N. A., Mc Neil, M. H., Norton, L. A., & Duell, R. C. (1974). The effects of tooth movement upon endodontically treated teeth. *The Angle Orthodontist*, *44*(3), 235–242. https://doi.org/10.1043/0003-3219(1974)044<0235:TEOTMU>2.0.CO;2	Endodontically treated teeth with a trauma history
3	Yoshpe, M., Kaufman, A. Y., & Einy, S. (2025). Effect of orthodontic treatment on traumatized teeth treated by regenerative endodontic procedure. *Angle Orthodontist*, *95*(2), 173–178. https://research.ebsco.com/linkprocessor/plink?id=68e5503f-1cd7-37b0-92a2-2a316866ebe0	Endodontically treated teeth with a trauma history
4	Singh, M., & Kaur, P. (1990). Temporary endodontics during active orthodontics. *Journal of the Indian Dental Association*, *61*(12), 279, 281, 283. http://www.ncbi.nlm.nih.gov/pubmed/2130099	Case report/series
5	Smidt, A., & Keinan, D. (2021). Accelerated healing of endodontically treated teeth with a periapical lesion as a result of orthodontic extrusion: evaluation and rationale. *Quintessence International (Berlin, Germany : 1985)*, *53*(1), 16–22. https://doi.org/10.3290/j.qi.b2091197	Case report/series
6	Suda, N., Kawafuji, A., & Moriyama, K. (2009). Multidisciplinary management including endodontics, periodontics, orthodontics, anterior maxillary osteotomy and prosthetics in an adult case with a severe openbite. *Orthodontic Waves*, *68*(1), 42–49. https://doi.org/10.1016/j.odw.2008.10.002	Case report/series
7	Vieyra, J. P. (2018). Nonsurgical orthodontic-endodontic treatment of a maxillary central incisor diagnosed with infected dens invaginatus (Oehlers’ type III) and associated apical periodontitis. *Endodontic Practice Today,* 12(1), 43-43.	Case report/series
8	Alharbi, M. (2020). Long-term Follow-up for Immature Teeth Treated with Regenerative Endodontic Procedures and Underwent Orthodontic Treatment. *European Endodontic Journal*. https://doi.org/10.14744/eej.2020.29591	Case report/series
9	Jawad, Z., Bates, C., Duggal, M., & Nazzal, H. (2018). Orthodontic management of a non-vital immature tooth treated with regenerative endodontics: a case report. *Journal of Orthodontics*, *45*(4), 289–295. https://doi.org/10.1080/14653125.2018.1501935	Case report/series
10	Yeap, C. W. (2023). Endodontic treatment of an immature maxillary lateral incisor with type II dens invaginatus in an orthodontic patient: A case report: The Bulletin of the Australian Society of Endodontology. *Australian Endodontic Journal*, *49*(2), 373–379. https://doi.org/10.1111/aej.12653	Case report/series
11	Liu, H., Hao, J., & Shen, Y. (2024). Partial regression of a healed periapical lesion in an endodontically treated premolar during orthodontic extrusion: A case report. *Journal of Dental Sciences*, *19*(3), 1894–1896. https://research.ebsco.com/linkprocessor/plink?id=5a823c96-b48b-3117-9a79-7890668ea0c7	Case report/series
12	Mendonca, M. R., Holland, R., & Almeida, M. H. C. (2000). Effects of orthodontic force on tooth endodontically treated. *Journal of Dental Research,* 79(5), 1141-1141.	Conference abstract
13	Consolaro, A., & Consolaro, R. B. (2013). Orthodontic movement of endodontically treated teeth. *Dental Press Journal of Orthodontics*, *18*(4), 2–7. https://doi.org/10.1590/S2176-94512013000400002	Literature review
14	Chaniotis, A. (2018). Orthodontic Movement after Regenerative Endodontic Procedure: Case Report and Long-term Observations. *Journal of Endodontics*, *44*(3), 432–437. https://doi.org/10.1016/j.joen.2017.11.008	Endodontically treated teeth with a trauma history
15	Natera, M., & Mukherjee, P. M. (2018). Regenerative Endodontic Treatment with Orthodontic Treatment in a Tooth with Dens Evaginatus: A Case Report with a 4-year Follow-up. *Journal of Endodontics*, *44*(6), 952–955. https://doi.org/10.1016/j.joen.2018.01.011	Case report/series
16	Chaniotis, A., & Chanioti, A. (2022). Long-term Complications of Previously Successful Regenerative Endodontic Procedures after Orthodontic Movement: A Report of 3 Different Complications after 4, 8, and 11 Years. *Journal of Endodontics*, *48*(7), 951–960. https://doi.org/10.1016/j.joen.2022.04.002	Case report/series
17	Keinan, D., Szwec, J., Matas, A., Moshonov, J., & Yitschaky, O. (2013). Applying extrusive orthodontic force without compromising the obturated canal space. *The Journal of the American Dental Association*, *144*(8), 910–913. https://doi.org/10.14219/jada.archive.2013.0208	Case report/series
18	Asnani, M. M., Jalaluddin, M., Goyal, V., Naqvi, Z. A., Gupta, B., & Sonigra, H. M. (2017). Assessment of the Effect of Orthodontic Treatment on the Periodontal Health of Endodontically Restored Tooth. *The Journal of Contemporary Dental Practice*, *18*(7), 587–590. https://doi.org/10.5005/jp-journals-10024-2089	Periodontal health not periapical health was assessed
19	Reddy, H., Singh, N., Vaghela, J., Sharma, S., Sachdeva, S. & Mishra, S. S. (2023). Effect Of Orthodontic Treatment On Endodontically Treated Tooth: A Clinical Study. *Journal of Pharmaceutical Negative Results*; 14 (2), 1338–1341.	Poor study quality outcome
20	Bud, E., Păcurar, M., Vlasa, A., Bica, C., Martha, K., Eșian, D., Bukhari, C., Lazăr, L., & Bud, A. (2021). Cone Beam Computed Tomography Study on the Incidence of Endodontic Filling Biomaterials Resorption in Patients With/Without Orthodontic Treatment. *Journal of Interdisciplinary Medicine*, *6*(2), 103–107. https://doi.org/10.2478/jim-2021-0023	Not related to research objectives
21	De Souza, R. S., De Souza, V., Holland, R., Gomes—Filho, J. E., Murata, S. S., & Sonoda, C. K. (2009). Effect of calcium hydroxide—based materials on periapical tissue healing and orthodontic root resorption of endodontically treated teeth in dogs. Dental Traumatology, 25(2), 213–218. https://doi.org/10.1111/j.1600-9657.2008.00758.x	Not related to research objectives
22	Asgary, S., & Talebzadeh, B. (2023). Impact of Orthodontic Treatment on Regenerative Endodontics in Immature Teeth: A Long-Term Case Study. CUREUS JOURNAL OF MEDICAL SCIENCE, 15(10). https://doi.org/10.7759/cureus.46953	Not related to research objectives
23	Mah, R., Holland, G. R., & Pehowich, E. (1996). Periapical changes after orthodontic movement of root-filled ferret canines. *Journal of endodontics*, *22*(6), 298–303. https://doi.org/10.1016/S0099-2399(96)80263-9	The lesion size was compered only with or without orthodontic tooth movement in vital or non-vital teeth, however, no results were reported on the changes in periapical lesion size after ortho treatment.
